# Telemedicine Expansion in Pediatric Gastroenterology in Response to COVID-19: Early Results of an International Physician Survey

**DOI:** 10.1097/PG9.0000000000000030

**Published:** 2020-12-23

**Authors:** Steven D. Miller, Jennifer A. Lee, Zachary Murphy, Jeremy Screws, Elizabeth A. Berg, Joseph A. Picoraro, Ayse P. Gurses, Jeannie S. Huang

**Affiliations:** From the * Division of Pediatric Gastroenterology, Hepatology, and Nutrition, Johns Hopkins University School of Medicine, Baltimore, MD;; † Department of Family Medicine, The Ohio State University Wexner Medical Center,; ‡ Division of Pediatric Gastroenterology, Hepatology,and Nutrition Nationwide Children’s hospital, Columbus, OH;; § Johns Hopkins University School of Medicine, Baltimore, MD;; ¶ Division of Pediatric Gastroenterology, Hepatology, and Nutrition, Children’s Hospital at Erlanger, Chattanooga, TN;; ‖ Division of Pediatric Gastroenterology, Hepatology and Nutrition, Columbia University Irving Medical Center,; # Pediatric Intestinal Rehabilitation Program, New York-Presbyterian Morgan Stanley Children’s Hospital, New York, NY;; ** Pediatric Gastroenterology Inpatient Services, New York-Presbyterian Morgan Stanley Children’s Hospital, New York, NY;; †† Armstrong Institute Center for Health Care Human Factors,; ‡‡ Anesthesiology and Critical Care Medicine, Johns Hopkins University, Baltimore, MD; and; §§ Rady Children’s Hospital and University of California San Diego, San Diego, CA.

**Keywords:** telemedicine, coronavirus, pandemics, surveys and questionnaires

## Abstract

The COVID-19 pandemic triggered an unprecedented expansion of telemedicine, leading to development of new workflows. We conducted a survey of telemedicine practice among pediatric gastroenterology practitioners on March 26, 2020. Responses were coded and analyzed. The survey garnered 33 responses. Most centers were 3 weeks into the implementation. The most commonly used telemedicine software was Zoom followed by FaceTime, telephone, and Epic software. Provider education was through online meetings, webinars, and tip sheets. Patient education was by nonclinical staff at the time of visit scheduling or tip sheets. A major barrier was the need for patients to enroll in an electronic portal. Two thirds of practices offered telemedicine to both new and return patients. Most sites billed based on time. This represents a record of the very early response of the pediatric gastroenterology community to the COVID-19 telemedicine expansion and can inform follow-up studies.

## Abstract 2

Supplemental Digital Content is available in the text.

**WHAT IS KNOWN/WHAT IS NEW**

**What is Known**

- In the past, telemedicine had limited use due to financial and regulatory barriers.
- The novel coronavirus triggered a massive expansion of telemedicine.

**What is New**

- Pediatric gastroenterologists frequently use Zoom for telemedicine visits.
- Most practices see both new and return patients by telemedicine.
- Many physicians bill for telemedicine based on time.

**COVID-19** is a novel coronavirus that began to circulate in Wuhan, China, in December 2019. The rapid dissemination of COVID-19 infection worldwide was declared a pandemic March 11, 2020.^1^ As of August 26, 2020, the virus infected over 24 million confirmed cases.^2^ The spread of coronavirus led governments across the world to recommend social distancing,^3,4^ with implementation of telemedicine^5^ to limit healthcare dissemination.^6^ Telemedicine capacity in the United States before the outbreak was limited in most geographic areas due to barriers including difficult to use technology, resistance to change, cost, and reimbursement.^7^ The mandate to expand telehealth in combination with relaxation of privacy standards^8^ and expanded reimbursement^9^ triggered a massive growth in telemedicine.

A consequence of rapid expansion of telemedicine in response to the pandemic is that hospitals/clinics may have been unable to follow standard design, planning, development, testing, and education protocols for deployment of electronic health record (EHR) technology.^10^ At the best of times, telemedicine implementation can lead to service fragmentation and technical failures.^11^ During this rapid expansion with higher patient acuity, limited provider experience with telemedicine,^12,13^ and implementation of unfinished telemedicine products and procedures, the risks and stakes were even higher.

The field of pediatric gastroenterology (Peds GI) is a useful system in which to study the rapid implementation of telemedicine in the face of COVID-19 because of its long history of multiinstitutional data sharing and collaboration,^14,15^ the existence of an active online community of EHR champions,^16^ and its status as a specialty that often works within larger hospital systems. The Peds GI telemedicine response can therefore serve as an exemplar of the telemedicine expansion. To understand telemedicine experiences among the Peds GI community, a brief survey on current telemedicine practice during the COVID-19 telemedicine expansion was sent out to the Peds GI community through a shared listserv. The results of the survey are presented below.

## METHODS

An eight question survey with semi-structured questions (See Table, Supplementar Digital Content 1, http://links.lww.com/PG9/A4) was sent out to the Peds GI listserv out of the University of Vermont. A short demographic follow-up survey was sent to respondents using SurveyMonkey (See Table, Supplementar Digital Content 2, http://links.lww.com/PG9/A5). Surveys were completed from March 26, 2020, to April 7, 2020. After the survey responses were received, a grounded theory approach was utilized to emergently define codes for free text responses, which were then organized into concepts.^17^ Two authors (SM and ZM) independently coded responses and a consensus approach was used to resolve differences. In order to assess the landscape of opinion, counts of each concept were quantified and particularly insightful comments were noted, with results presented below. The research was approved by the Johns Hopkins Medicine IRB.

## RESULTS

### Demographics

Respondents comprised a broad mix of geographic locations including all census regions of the United States,^18^ as well as Canada (n = 3, 12%), the United Kingdom (n = 1, 4%), and Australia (n = 1, 4%) (Table 1, n = 26 for demographic practice survey). There was some representation from private practice (n = 3, 12%), although most respondents came from academia (n = 17, 65%). Most practices were urban (n = 17, 65%), although some were suburban (n = 6, 23%) or mixed (n = 3, 12%). The overwhelming majority of respondents used Epic (n = 19, 76%), but there was some representation of other EHR’s. All practices were very early in their telemedicine implementation (range 0–4 weeks).

**TABLE 1.** Demographics of Cohort

Demographic Table		Number (%)
Coronavirus hot spot		
	Yes	9 (34.6)
	No	14 (53.8)
	Unsure/other	3 (11.5)
Number of practicing providers (MD, PA, NP)		
	1–5	10 (38.5)
	6–10	7 (26.9)
	11–20	6 (23.1)
	>20	3 (11.5)
Practice type		
	Private	3 (11.5)
	Academic	17 (65.4)
	Other	6 (23.1)
Practice location		
	Urban	17 (65.4)
	Suburban	6 (23.1)
	Rural	0 (0)
	Mixed	3 (11.5)
Electronic health record		
	Epic	19 (76)
	Cerner	2 (8)
	Allscripts	1 (0)
	Other	3 (12)
Region (based on US census divisions)^19^		
	US-South Atlantic	11
	US-Middle Atlantic	5
	US-East South Central	3
	Canada	3
	US-East North Central	3
	US-Pacific	3
	US-New England	2
	Other country	2
	US-Mountain	1
	US-West South Central	1
Weeks since COVID-19 telemedicine implementation (median, range)		3 (0–4)

MD indicates medical doctor; PA, physician assistant; NP, nurse practitioner.

### Technology

**FIGURE 1.**
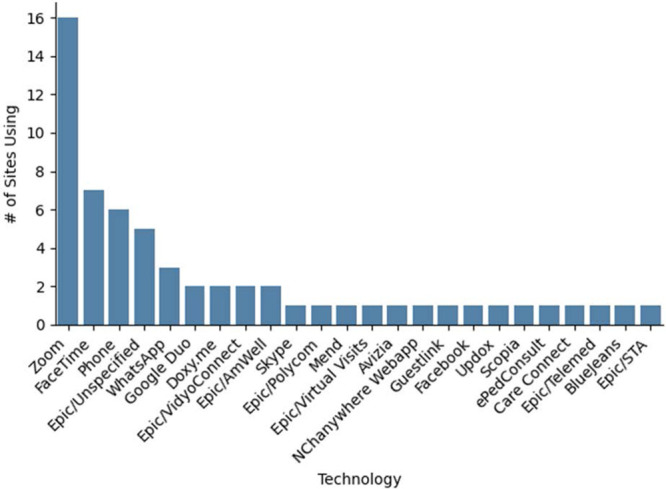
Telemedicine software used among survey respondents presented in order from most to least frequent.

The initial survey garnered n = 33 unique responses. Figure 1 presents the telemedicine technology used by respondents. Participants could provide more than 1 response. The most used applications were Zoom (n = 16, 48%) followed by Facetime (n = 7, 21%), although in many cases, these were cited as an alternative to a primary application. Phone (n = 6, 18%) and Epic/Haiku (n = 5, 15%) were the third and fourth most commonly used applications. Most applications were used only by one or two responding institutions. Providers reported a variety of problems with telehealth software including dropped telemedicine calls and lack of a back-up option for patients not already enrolled in the online patient portal.

### Provider Education

Physician education for telemedicine varied widely. The most commonly reported responses were online meetings (n = 16, 48%), webinars (n = 11, 33%), and tip sheets (n = 11, 33%). Several institutions reported having a division expert (n = 5, 15%) who provided tech support and a number of respondents reported referring to an institutional intranet site (n = 4, 12%). Some providers (n = , 6%) brought up that email communication were sometimes unclear, with one provider stating, “There were a few emails that went out, but they were confusing to many.”

### Patient/family Education

Patient/family education for telemedicine was similarly heterogeneous across the cohort. The most common educational modality was instruction by nonclinical staff before the appointment (n = 16, 48%), with large numbers also reporting sending emails or messages through the electronic health record (EHR) portal (n = 11, 33%) or educating patients at the time of scheduling (n = 11, 33%). Involvement of other staff members was also noted at many centers, with reported use of nursing staff (n = 5, 15%), medical students (n = 4, 12%), and physicians (n = 2, 6%). Some respondents (n = 3, 9%) cited enrollment in a patient portal as a barrier to patient participation in EHR-based telemedicine visits. One center reported that the requirements for proxy access by parents had been temporarily waived, and another center reported that their endoscopy nurses were devoted full time to signing patients up for the portal. Some providers (n = 3, 9%), reported that patients often were unprepared for the telemedicine visit, downloading telemedicine software at the time of the visit and causing delays.

### New vs Return Patients

There was a split among respondents regarding types of patients offered telemedicine services. Most practices (n = 21, 63%) reported offering telemedicine visits to both new and return patients, while others offered telemedicine visits to return patients and preferred to see new patients in person (n = 11, 33%). Several providers (n = 5, 33%) added the caveat that telemedicine may not work for acute complaints such as weight loss or gastrointestinal bleeds. One respondent reported seeing all patients initially by telemedicine but getting patients in for a physical within 1 week if needed. Some providers (n = 2, 6%), reported that providers in their group split in-person duties, with each provider coming to clinic once weekly to see any patients who needed physical exams. One provider reported feeling, “very, very stressed” about triaging patients to in-person versus telemedicine visits due to concerns about liability.

### Telemedicine Adoption

The adoption of telemedicine varied across respondents. A good proportion of centers (n = 14, 42%) reported seeing most patients by telemedicine, although many centers reported that their visit numbers were far lower than pre-COVID levels. Some centers reported still seeing most patients in person (n = 5, 15%), and others had not yet started telemedicine services at the time of the survey (n = 11, 33%). One respondent to the survey noted, “One of the few bright spots of this crisis is that we can now do telehealth going forward.”

### Billing

In terms of billing, the most common response was that providers did not know how to bill or were unsure about how billing using new telehealth billing codes would compare to pretelemed billing cycles (n = 21, 63%). Several providers reported billing based on time (n = 11, 33%) and some reported that they were flagging all of their telehealth charges for financial staff to review and edit later (n = 4, 12%). Many respondents reported concerns about decreased revenues (n = 6, 18%) or worries that charges would be denied (n = 4, 12%).

### Physical Exam

A question pertaining to physical exam was only included in the follow-up survey. A small number (n = 5, 26%) document in their note that no physical exam was performed. The majority (n = 17, 65%) report either a minimal exam or include some abdominal exam such as reporting tenderness upon parent’s palpation or patient jumping. Some respondents (n = 7, 27%) reported doing an extended visual exam of multiple systems such as general appearance, HEENT, head, respiratory, abdomen including parent palpation, neurological exam, and skin.

## DISCUSSION

This manuscript presents early results of real world telemedicine implementation during the COVID-19 pandemic. The survey reflects views of practicing pediatric gastroenterologists and may not be reflective of organizational intent. Respondents were broadly distributed across the United States with some representation from abroad and a predominance of smaller practices, reflecting the typical demographics of Peds GI practice. These results represent a microcosm of the wider world of rapid implementation of technology and provide valuable insight into the heterogeneity of early implementation strategies and training practices for patients and providers. This manuscript presents data representing an initial limited snapshot of telemedicine practice from a small subset of Peds GI doctors that can inform the development of practice standards and be utilized to track changes over time.

In the United States, before the COVID-19 pandemic, audio/visual telemedicine software was required to have a business associate agreement with HIPAA compliant organizations. At the beginning of the pandemic, the Office for Civil Rights lifted regulations to allow for the rapid response.^8^ Zoom (https://zoom.us/healthcare), which has the capacity to be HIPAA compliant with a business associate agreement, was the most widely used software option, with overall positive reports about its functionality. While there were no follow-up questions to understand which type of Zoom contract was utilized at each institution, it can be integrated with EHRs. Recent concerns about vulnerability of the Zoom software to hackers^17^ have potential to alter rates of use. Follow-up surveys will yield insights into how the technology and practices adjust to real world experience.

Our survey did not establish the most effective methodologies for telemedicine training during a pandemic or otherwise, but it did show that online meetings and video trainings were most commonly used followed by tip sheets and divisional experts. Despite variability in training, there were no complaints of inability to utilize the software. Further investigation can help inform future telemedicine implementations.

Patient telemedicine training was mostly provided by non-clinical staff prior to visits and through emailed messages, with several providers reporting that this prevented technical problems from getting in the way of doctor-patient interaction. Providers nonetheless reported that some patients had technical difficulties that made it difficult to complete a telemedicine encounter. Our survey was notably missing the voices of patients and families to provide insight into what has worked and not worked for them in comprehending this telemedicine expansion and utilizing the accompanying technologies, and additional studies are needed.

Prior to the Centers for Medicare and Medicaid emergency waivers, telemedicine appointments required an established relationship between the patient and provider. In our survey, there was variability around the use of telemedicine for new versus return visits. In current practice, the decision to see new patients by telehealth may occur on a per-provider or per-institution basis in response to local factors such as availability of urgent in-person follow-up visits, prevalence of COVID-19 in the population, and provider preference. Survey respondents were interested in getting guidance through the development of standardized practices as to which chief complaints or diagnoses would be appropriate for telemedicine. Importantly, understanding the clinical outcomes for telemedicine encounters compared with in-person visits will be vital to determining the long-term role of telemedicine visits in the continuum of care.

The highly heterogeneous answers about the numbers of patients being seen through telemedicine may represent the early phase at which this survey was conducted. Similarly, the lack of clear response on billing may be due to very few bills having been submitted at such an early stage in the COVID-19 telemedicine expansion. The lack of clarity on billing from providers is likely in part related to the rapidly shifting payment infrastructure that will continue to evolve over time. This survey establishes a practice baseline upon which follow-up studies can build.

This study had several limitations. The small number of responses limits the generalizability of the findings. The survey was carried out very early on in the pandemic, and one-third of respondents had not begun seeing patients by telemedicine, further reducing the representation of those who had actually implemented telemedicine. The method of collecting responses on the listserv could have introduced sampling bias from a population that self-selected for interest in telemedicine or social desirability bias in the content of responses. Follow-up studies of the telemedicine implementation in Peds GI should address these methodologic limitations.

There is potential for this to be a transformational moment with regard to broad use of telemedicine, not just to handle emergent situations such as COVID-19 or natural disasters,^18^ but for everyday clinical practice. The COVID-19 crisis offers the community of Peds GI and all other health practitioners an opportunity to test drive telemedicine technology, develop best practices, and work together to establish a useful place for telemedicine in our clinical practice in the future.

## ACKNOWLEDGMENTS

We thank the following survey respondents for their participation: Thirumazhisai Gunasekaran, Kevin Watson, Cary Sauer, Marc Tsou, Sivan Kinberg, Steven Liu, Ian Leibowitz, Lizsa Tan, Esther Israel, David E. Deutsch, Daniel DiMeo, Howard Baron, Kathy Schwarz, Rupinder Gill, Jason Silverman, Daphne Say, Iona Monteiro, Albert Chan, Runa Watkins, Michael A. D’Amico, Pamela Goodman, Lourdes Herrera.

## Supplementary Material
