# Supplementary material for: Telemedicine Expansion in Pediatric Gastroenterology in Response to COVID-19: Early Results of an International Physician Survey
Source: JPGN Rep. 2020 Dec 23;2(1):e030. doi: 10.1097/PG9.0000000000000030 (PMC10191549; doi:10.1097/PG9.0000000000000030)
Supplement: Supplementary file 1 [file pg9-2-e030-s001.pdf]

Supplemental digital materials 1: Provider survey questions

1. What technology are you using for telemedicine visits and how is it working?
2. How are providers being educated about telemedicine visit mechanics?
3. How are patients/families being educated about telemedicine visit mechanics?
4. Are large numbers of patients being seen by Telemedicine yet?
5. What are you doing about new vs. return patients?
6. How are you accomplishing scheduling and rescheduling?
7. How is billing going?
8. Other comments?
