# Supplementary material for: Telemedicine Expansion in Pediatric Gastroenterology in Response to COVID-19: Early Results of an International Physician Survey
Source: JPGN Rep. 2020 Dec 23;2(1):e030. doi: 10.1097/PG9.0000000000000030 (PMC10191549; doi:10.1097/PG9.0000000000000030)
Supplement: Supplementary file 2 [file pg9-2-e030-s002.pdf]

Supplemental digital materials 2: Demographic questionnaire

**Demographic Questionnaire**		
**Question**	**Response Type**	**Total Responses**
What is your email address (for matching with survey responses?)	Free text	28
What is the clinic/hospital where you work (for matching with survey response)?	Free text	28
Do you wish to be acknowledged in the manuscript?	Yes, No	28
Is your clinic/hospital in the location of a coronavirus hot spot?	Yes, No, Unsure, Other (please specify)	28
About how many providers (MD, DO, NP, PA) are in your pediatric GI practice?	Free text	28
Are you in private or academic practice?	Private, Academic, Other (please specify)	28
How would you describe the location of your practice?	Urban, Suburban, Rural, Other (please specify)	28
What electronic health record vendor do you use?	Epic, Cerner, Allscripts, Other (please specify)	27
How many weeks are you into your telemedicine implementation? (The start date is when you began converting in-person to telemedicine visits)	0-20	28
About how long has your practice been conducting at least some telemedicine visits (specify number of weeks or months or years)?	Free text	28
